# Identifying mechanisms of change in a magic-themed hand-arm bimanual intensive therapy programme for children with unilateral spastic cerebral palsy: a qualitative study using behaviour change theory

**DOI:** 10.1186/s12887-020-02246-y

**Published:** 2020-07-31

**Authors:** Daisy Fancourt, Jaeyoung Wee, Fabianna Lorencatto

**Affiliations:** 1 Department of Behavioural Science and Health, University College London, 1-19 Torrington Place, London, WC1E 7HB UK; 2 Centre for Behaviour Change, University College London, London, UK

**Keywords:** USCP, Behaviour change, Psychosocial, Barriers, Enablers, Mechanisms, Children, Arts, Magic

## Abstract

**Background:**

There has been much research into how to promote upper-limb skills to achieve functional independence in children with unilateral spastic cerebral palsy (USCP). One researched intervention is the Breathe Magic programme, which follows the protocol of hand-arm bimanual intensive therapy (HABIT) whilst, incorporating magic tricks to develop children’s motor skills and bimanual skills. However, whilst research has found the programme to be effective, there has been little consideration of how the intervention leads to a positive outcome: what the psychological, social and physical mechanisms of action are.

**Methods:**

Qualitative semi-structured interviews with 21 children with USCP who participated in the Breathe Magic HABIT intervention, and focus groups with 17 parents and/or carers were undertaken. Analysis was conducted through the lens of the COM-B behaviour change model using a combined deductive framework and inductive thematic analysis. Reliability of coding was confirmed through random extraction and double coding of a portion of responses and the calculation of inter-rater reliability.

**Results:**

Breathe Magic brings about change and positive outcomes by increasing children’s psychological and physical capabilities, providing social opportunities, and enhancing reflective and automatic motivation. Additionally, a number of enablers to engaging in the intervention were identified, particularly under psychological capabilities, social opportunities and both reflective and automatic motivation. Very few barriers were raised; those that were raised were of relatively low frequency of reporting.

**Conclusions:**

By conducting a theory-based qualitative process evaluation, this study demonstrated the mechanisms of change behind the Breathe Magic HABIT intervention for children with USCP. Breathe Magic was found to be a well-structured combination of intended and unintended mechanisms of change. Overall, the success of Breathe Magic was observed through not only its intended mechanisms to enhance hand skills, but also through unintended psychological improvements in children’s hand function, as well as social and motivational benefits resulting from interaction between children and parents.

## Background

Unilaterial spastic cerebral palsy (USCP, or “hemiplegia”) is a neurological condition affecting around 1.77 to 2.11 in every 1000 live births [1, 2], in which one side of the body is paralysed due to damage to or abnormalities in the brain [3]. Primary symptoms include impaired motor function, speech and balance [4–8], which can lead to difficulties in managing daily tasks independently. In particular, children with a unilateral impairment tend to avoid using the affected limb, even if they are suffering mild functional loss [9]. This can lead to increased impairment in the affected limb [10], including reduced motor control, motion, and skeletal maturation [11]. Poor movement of the affected hand can also reduce the performance of a non-affected hand [9]. Alongside these physical symptoms, children with USCP can also experience behavioural problems, learning and visual difficulties, and developmental delay [4, 6–8]. Improving the use of affected hands is, therefore, critical to the functional and emotional development of children with USCP.

There has been much research into how to promote upper-limb skills to achieve functional independence in children with USCP, with a number of interventions identified [12]. Interventions include botulinum toxin injections, Constraint-Induced Movement Therapy (CIMT; which involves therapy using the most-affected limb, while restricting the use of the unaffected limb) [13], and Hand-Arm Bimanual Intensive Therapy (HABIT; another intensity therapy focusing on the improvement of coordination using both hands through functional activities) [14]. Specifically, HABIT usually consists of repetitive, task-specific training based on neuroscientific literature with between 30 to 90 h of high intensity practice in a group setting [15].

One form of HABIT therapy that has proved popular in recent years is the Breathe Magic HABIT programme. This intervention is similar to a number of other programmes that have, in recent years, incorporated theme-based approaches in order to support child engagement and outcomes [16, 17]. Further, it builds on research suggesting the importance of goal setting in the achievement of improvements in gross motor function [18]. The Breathe Magic intervention follows the HABIT protocol whilst, incorporating magic tricks involving cards, cups, balls, and rubber bands to develop children’s motor skills and bimanual skills. Research into the programme found that the Breathe Magic programme increased usage of children’s affected hands by 61% sustained after a follow-up 3 months later, regardless of age, severity of movement restriction, or nationality [19]. This was accompanied by improvements in activities of daily living 3 months following the intervention [20]. Similarly, a further study showed that 92% of children had improvements in bimanual ability and 75% of them maintained their improvement after a follow-up 6 weeks later [21]. They also found the level of activation in the affected hemisphere within the brain of the participants increased by approximately 34% [21]. Notably there was also a significant increase in the White Matter (WM) integrity in the corpus callosum and corticospinal of the participants, which has a high correlation with better hand function [21]. However, it should be noted that another study using a modified protocol of Breathe Magic (but unaffiliated to the Breathe Magic programme) did not find improvements in bimanual ability [22], and studies specifically comparing the effect size of Breathe Magic to non-magic HABIT interventions have not been carried out.

Nevertheless, Breathe Magic remains a popular treatment option for children and their families. But to date there has been little consideration of *how* the intervention leads to any positive outcomes: what psychological, social and physical processes that enable an improvement in bimanual ability. These processes are commonly referred to as “mechanisms of change” or “mechanisms of action” (hereafter simply called “mechanisms”) [23]. To date, a few studies have identified factors within magic-based HABIT programmes that could be considered mechanisms. For example, a qualitative study found that novelty and specialness of the magic approach were key components in making magic-themed HABIT and helped to increase self-belief and overall motivation [24]. Further, a quantitative study specifically of Breathe Magic identified improvements in children’s self-confidence [19]. However, identification of further mechanisms remains to be undertaken. Further, it is unclear how many of these mechanisms are specific to the magic component of the Breathe Magic intervention, and thus potentially unique to this intervention, and how many of the mechanisms are in fact common to other interventions for children with USCP. Understanding these mechanisms through process evaluations is critical for identifying how and why interventions work and to enable their replication [25].

Best practice guidelines for process evaluations advocate for the use of theories to understand data [25]. Explicit use of theory in evaluating interventions has several benefits. First, theory can be used to inform the understanding and delivery interventions by identifying constructs (key concepts in the theory) that are hypothesised to be causally related to behaviour and are therefore appropriate targets for the intervention. Changing constructs that cause behaviour will, theoretically, lead to behaviour change [26]. Second, collecting empirical data within a theoretical framework facilitates the accumulation of evidence of effectiveness across different contexts, populations and behaviours, such that comparisons could be made (i.e. between Breathe Magic and other interventions). Third, using theory can aid understanding of why interventions are effective or ineffective by facilitating an understanding of mechanisms of change [27].

Although use of theory to design and evaluate interventions is now widely accepted and advocated for, it is very rarely done, particularly for process evaluations [28]. As Breathe Magic is a complex intervention that aims to bring about change in behaviours and targets a range of physical and socio-emotional outcomes, it is relevant and appropriate to apply a behavioural science lens. Whilst there are over 80 identified behaviour change theories, many of these theories are partial and/or overlap. There is also limited guidance for selecting one theory over another. There have thus been efforts to integrate behavioural theories into a minimum set of constructs representing the range of influences on behaviour. COM-B is one such integrative model of multiple different behaviour change theories (COM-B) proposes that Behaviour change (B) is underpinned by three interacting factors: Capability (C), Opportunity (O) and Motivation (M) [29]. Each of these can be further broken down into two factors, representing six domains of behavioural influences in total. Capability refers to both the *psychological* and *physical* capacity to engage in a physical process, including having necessary knowledge and skills. Opportunity refers to factors that lie outside an individual’s control and influence behaviour: *physical opportuni*ty refers to the physical environment, while *social opportunity* is afforded by the cultural setting that directs individuals’ thinking. Motivation encompasses both *reflective* processes, involving analytical decision-making and plans, and *automatic* processes, involving habitual processes and emotional responses. Mechanisms can thus be conceptualised and understood within this behavioural framework [23].

COM-B has been applied in a number of existing studies relating to health and non-health behaviours (e.g. Alexander, Brijnath, & Mazza, 2014 [30]; Barker, Atkins, & de Lusignan, 2016 [31]; Jackson, 2014 [32]), but has not previously been applied to Breathe Magic or any other HABIT interventions for children with USCP. Therefore, this study used the lens of COM-B to identify the mechanisms of change that led to improvements in children with USCP who took part in the Breathe Magic HABIT intervention. We considered the overall behaviour change of the intervention to be ‘improved bimanual ability’. It also had a secondary aim to identify any factors that act as barriers or enablers to engaging with the intervention, and therefore influence whether change can be achieved.

## Methods

### Design

This was a qualitative semi-structured interview study involving children with USCP who participated in the Breathe Magic HABIT intervention, and focus groups with their parents and/or carers.

### Participants

Participants were recruited from the 2016–2017 Breathe Magic HABIT Programme in London. Inclusion criteria for children to participate in the programme were (i) aged 7–19, (ii) a diagnosis of congenital or acquired USCP/hemiparesis, (iii) attending mainstream school or enrolled at an equivalent educational institute, and (iv) a desire to learn magic tricks. Exclusion criteria are moderate to severe cognitive impairments, overt behavioural disorders, seizure disorders not well-controlled by medication, severe dystonic presentation, Brachia I Plexus injuries, and bilateral presentations of USCP. A total of 27 children were found to be eligible and participated in the programme in 2016, of whom 21 were present at the 6 month follow-up session. We approached all of these 21 children face-to-face at the session. All agreed to be involved in interviews for this study, and all consented to participate. We also approached all of their parents and/or carers face-to-face, of whom 17 of the 21 consented to be involved in focus groups. The remaining 4 declined due to lack of availability.

The children were aged 7–17 years (average age 11) and a mixture of boys (*n* = 9; 43%) and girls (*n* = 12; 57%). Severity of movement difficulties at baseline had been assessed using the Manual Ability Classification System (MACS), which ranks a person’s ability to handle objects in important daily activities. 29% of the children had scored as level I, 48% as level II, and 23% as level III, which indicate a range of abilities from general success in performing manual tasks but with some problems in speed and/or accuracy, through to difficulty in handling objects including slow performance and limited achievement requiring support and/or modified activities. Due to the small numbers of children in the programme and the possibility for participant identification, further detail on the specific demographics of children was not approved for publication by the ethics committee.

### Intervention

Breathe Magic is a well-researched, intensive bimanual therapy programme where children aged 7–19 with USCP learn specially selected magic tricks designed to help improve the use of their affected hand and arm. Magic Circle magicians work alongside occupational therapists to teach magic tricks and performing skills to develop motor and bimanual skills and enhance independence, confidence and self-esteem in a 1-to-1 group therapy setting. The programme is designed to appear as a non-clinical, non-education intervention. Activities are tailored to suit each young person’s particular needs. The programme begins with a 10-day intensive intervention over two consecutive weeks known as the Breathe Magic Camp. Camps are followed by 3 bi-monthly full-day Breathe Magic Clubs over the following six months. Clubs encourage participants to continue practicing the movements at home while helping children and their parents to build strong peer-support networks, which in turn promotes the sustainability of the programme. A total of 78 h of intensive therapy and 90 h of total clinical time is delivered over the course of the programme.

### Procedure

For this study, children participated in semi-structured face-to-face interviews lasting approximately 15 min while parents took part in two focus groups of 7 and 10 participants lasting approximately 1.5 h. These were both conducted at the final Breathe Magic Club (6 months following the end of the Breathe Magic Camp) by a researcher who had not been involved in any of the previous studies of the intervention and was not known to the participants. Nobody else was present at the interviews or focus groups. We chose to speak with parents and children separately to allow parents more freedom in discussing theirs and their children’s experiences honestly without concern over how their children would interpret any comments made. Further, we spoke to children individually rather than in a group as many of the children had difficulties affecting speech, so individual interviews allowed each child space and time to make their points. Both interviews and focus groups involved broad questions on their experience of the programme and specifically any physical, psychological and social changes children or parents had observed as a result of participating (see Appendix 1 for topic guides). Interviews and focus group discussions were audio-recorded and transcribed verbatim, with names changed to maintain anonymity and confidentiality.

### Analysis

Analysis was conducted through the lens of the COM-B behaviour change model. This method of analysis was selected prior to any analysis of the data taking place.

Transcripts were analysed in 4 phases, using a combined deductive framework and inductive thematic analysis approach [33]. Phase 1 involved familiarisation with the transcript and joint coding of one transcript by two researchers (FL, JW), to help develop a codebook. Neither researcher was involved in the delivery of the intervention, nor had any links to the organisation delivering. Phase 2 involved deductive framework analysis using the COM-B model as a coding framework. Participant responses were assigned to the COM-B domain they were judged to best represent. Where applicable, a response could be coded to more than one domain. Phase 3 involved inductive thematic analysis. Similar participant responses coded to the same COM-B domain were grouped together, and a theme label inductively generated. Theme labels summarised the role that COM-B domain played in bringing about change as a result of participating in the intervention, and/or barriers and enablers to change and engagement with the intervention. Phase 4 involved the identification of the key COM-B domains that were most influential mechanisms of change. Following the methods of Graham-Rowe et al. (2018) [34] and Atkins et al. (2017) [33], three factors were considered when identifying key domains. First, the frequency of a theme occurring across the interviews was counted, with each speaker counted once within each theme to generate a frequency count. Second, the degree of elaboration for each domain was calculated in terms of the number of themes and sub-themes inductively generated for each COM-B domain. Third, expressed importance was assessed based on whether participants qualitatively expressed in the interviews/focus groups that the particular theme/domain was an important influence on their behaviour and outcomes.

To ensure reliability of deductive coding to the COM-B domains, 10% of the extracted participant responses across transcripts (*n* = 227 in total) were randomly selected and independently coded by two researchers not involved in the intervention nor with any links to the organisation delivering the intervention. Inter-rater reliability was assessed using Cohens kappa. Participants did not receive or comment on the transcripts.

## Results

**Fig. 1:**
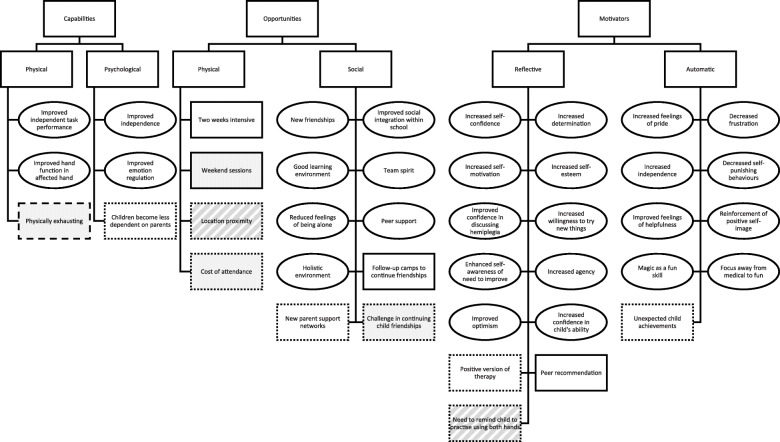
Mechanisms of change (oval box), enablers (white rectangle), barriers (grey rectangle) or both enablers and barriers (striped rectangle) to engagement with the Breathe Magic programme amongst children (___ outline) and parents (. . . . outline) or both (_ _ _ outline)

In total, 227 quotes were extracted and synthesized into 42 themes (Fig. 1). The kappa coefficient for inter-rating coding reliability was 0.874, indicating high reliability [35, 36]. Mechanisms of change were identified within all 6 of the COM-B components for both children (Table 1) and for parents (Table 2). Identified themes within each COM-B domain, their frequency, whether they are barriers/enablers/or both to change, and supporting quotes are presented in Table 1 and Table 2 for children and parents respectively.

**Table 1:** Summary of COM-B Themes classified as Mechanisms, Enablers and Barriers for Children

COM-B component	Identified Theme	Mechanism/ Barrier/ Enabler	Frequency (n transcripts)	Interviewees (Parents/Child, n)	Described as important (Yes/No)	Sample Quote
Physical Capability	Breathe Magic enables children to independently perform physical day-to-day tasks	Mechanism	25	Child (15) Parents (10)	No	Six months ago, I couldn’t tie a shoelace, now I can. Six months ago, I couldn’t even flap my hand. Now I can. (Child)
	Breathe Magic improves function in children’s affected hand	Mechanism	12	Child (7) Parents (5)	No	I can do pinch and I can move my hand more, and I’ve got this exercise I do. And then hold it for 30 s. (Child)
	Participating in Breathe Magic can be exhausting for children and parents	Barrier	2	Parent (2)	No	We would get some challenges sometimes and she would be fatigued and I was exhausted. (Parents)
Psychological Capability	Breathe Magic develops children’s independence	Mechanism	1	Parents (1)	No	He′s calmer in himself and will just sit there, maybe not ask for help. (Parents)
	Breathe Magic makes children to become capable of regulating emotion	Mechanism	1	Child (1)	No	I just used to go up to my bedroom sometimes every once in a while and just cry. I sometimes do that now, but I know how to get it better and stuff. Yeah. (Child)
Physical Opportunity	Weekend session is not favoured by children	Barrier	1	Child (1)	No	The thing I don’t like, it’s on a Saturday, it’s when I do my football. (Child)
	Breath Magic has two weeks back-to-back intense practice	Enabler	1	Parents (1)	No	and the fact that it is two weeks back-to-back, that works because it’s just so intense, it’s all about the two handed and I know you won’t (Parents)
Social Opportunity	Breathe Magic creates opportunity to form new friendships	Mechanism	17	Child (15) Parents (2)	No	I’ve made a lot of new friends and loads of friends and stuff. And I get to socialise a lot, because at school I don’t really socialise. I’m a bit shy. (Child)
	The Breathe magic team, magicians and mentors provide good learning environment	Mechanism	6	Child (1) Parents (5)	No	I like the teachers, the magic trainers. Especially Ed. … … … ..They give you enough time to do the trick, and if you get stuck they can help you. (Child)
	Breathe Magic creates a team spirit for children	Mechanism	4	Parents (4)	Yes	It was about a bunch of kids coming together for magic tricks … because they were, like you said, they were all in the same boat. (Parents)
	Social environment makes children feel that they are not alone	Mechanism	4	Child (2) Parents (2)	No	It gives them that little boost that I’m not on my own, I′ not the only person who’s got hemi.”. That helped them to see that socially, it’s kind of like you’re not alone. (Parents)
	Breathe Magic provides environment where children help each other	Mechanism	1	Child (1)	No	Q. What was the favourite thing about the camp. Ans. I like how people help people at different difficult times. Say if someone was stuck on something, some people might help them. (Child)
	Breathe Magic creates opportunity to see old friends	Enabler	1	Child (1)	No	Favourite thing about the camp - Ans. Probably seeing old friends again. (Child)
	Breath Magic improves integration with other children in mainstream schooling	Mechanism	2	Parents (2)	No	So she is, now, entirely immersed in mainstream education, whereas since nursery, she’s had an education plan and we’ve had to go in and targets and all that sort of stuff but she’s completely
	The holistic experience of Breathe Magic provides an engaging environment	Mechanism	3	Child (1) Parents (2)	Yes	And in terms of the best thing about the camp, I genuinely can’t think of anything that you could do to improve it. It’s the total sum of the different things, it’s the activities, it’s the people, it’s the environment, it’s everything (Parents)
Reflective Motivation	Breathe Magic increases children’s self-confidence	Mechanism	18	Child (2) Parents (16)	No	The confidence for Alex from the first day 10 days later is just through the roof (Parents)
	Breathe Magic makes children determined	Mechanism	13	Child (8) Parents (5)	No	That I can at least come away thinking it’s not like a terrible, terrible thing. You know I can try and I can try. I might not get it, but I can still give it a go. (Child)
	Breathe Magic increases children’s self-motivation	Mechanism	7	Parents (7)	No	she kind of became really confident and motivated with that side of it as well, which is good. (Parents)
	Breathe Magic increases children’s self-esteem	Mechanism	5	Child (1) Parents (4)	Yes	the best thing to come out of the camp is Erin’s self-esteem. (Parents)
	Children who participated in Breathe Magic would recommend participating the intervention to others	Enabler	4	Child (4)	No	Q. What would you say to somebody who was thinking about coming on this camp, someone else with USCP? Ans. Yes I would probably say something nice like just about coming in to the camp. (Child)
	Breathe Magic enables children to feel comfortable talking about their condition with others	Mechanism	3	Parents (3)	Yes	One of the ground-breaking things for her was that she was the confident to tell people about her USCP (Parents)
	Breathe Magic increases children’s willingness to try new things	Mechanism	2	Parents (2)	No	he was much more willing to give everything a go. (Paremts)
	Breathe Magic increase children’s self-awareness that they need to improve	Mechanism	2	Parents (2)	No	this is the self awareness that he should try (Parents)
	Breathe Magic increases child agency	Mechanism	1	Parents (1)	No	Q. it sounds like a lot of you have found that it was more self driven during those two weeks? Ans. Yes” (Child)
	Breathe Magic makes children to become optimistic about the future	Mechanism	1	Parents (1)	No	And then start looking negatively at herself and not wanting to be here, it’s quite hard for us because it’s been a very, very positive thing up until now … ..she, up to a few weeks ago, looked to the future with great optimism (Parents)
Automatic Motivation	Breathe Magic reinforces children’s positive feeling about themselves	Enabler	10	Child (9) Parents (1)	No	They (Magic Camp) won’t do stuff for you. You have to do it all by yourself. Because they push you to feel good about yourself. (Child)
	Magic is a fun skill for children to learn	Mechanism	8	Child (7) Parents (1)	No	I guess just … This might seem a bit obvious, but just learning the magic and just practise it, practising, and trying and just learning new tricks. I like it all really. It’s a nice feeling just doing magic and, yeah, and just have magic, yes. (Child)
	Breathe Magic makes children to feel proud of their achievement	Mechanism	6	Children (5) Parents (1)	Yes	Proud, to be honest. Like it was a sense of ... but at the same time it was kind of like, “Oh I can do this now.” It was kind of surreal because I was just like slowly progressing and I was watching myself do it (Child)
	Breathe Magic makes children become less frustrated	Mechanism	4	Parents (4)	No	The frustration was reduced. (Parents)
	Breathe Magic is not seen as therapy but fun practice to learn Magic	Mechanism	2	Parent (2)	Yes	that’s the important thing, isn’t it, that it’s therapy without being therapy (Parents)
	Breathe Magic makes children feel more independent	Mechanism	1	Child (1)	No	It makes me feel more independent. (Child)
	Breathe Magic helps children discontinue self-punishing behaviours	Mechanism	1	Child (1)	No	Alex had some quite negative physical behaviours towards himself because of how he felt before the camp. He′d sort of bite himself, hit himself, call himself ridiculous, directly because of the USCP and what he couldn’t do at any given time and I haven’t actually seen any of that, which is marvellous (Parents)
	Breathe Magic makes children feel helpful	Mechanism	1	Child (1)	No	It’s good to be able to feel like I can help in some way I guess. (Child)

**Table 2:** Summary of COM-B Themes Classified as Enablers and Barriers for Parents

COM-B component	Identified Theme	Mechanism/ Barrier/ Enabler	Frequency (n transcripts)	Interviewees (Parents/Child, n)	Described as important (Yes/No)	Sample Quote
Physical capability	Participating in Breathe Magic can be exhausting for children and parents	Barrier	2	Parent (2)	No	We would get some challenges sometimes and she would be fatigued and I was exhausted. (Parents)
Psychological capability	Breathe Magic helps parents to take a step back to allow the child to become more independent	Enabler	5	Parents (5)	No	just step back right from today and I had to say to myself emotionally because, to be clear and to just prepare myself where I’m going to step back and from the day, just stepping back just helped to give them the opportunity as well (Parents)
Physical opportunity	The location of Breathe Magic is (not) difficult to access	Barrier/ Enabler	3	Parents (3)	No	Because were offered some of these (Breath) services based on where you live and they are broad (Parents-Enabler) really mean like Heathrow because you can take direct Trains there, or you could drive there because central London is just so congested and when you have little ... kids, it’s just too much. The journey just tired them out as well. So, if it’s a simpler journey it would be much easier for the child (Parents-Barrier)
	Cost of attending the Breathe Magic is high	Barrier	2	Parents (2)	No	while we were doing it but then finding accommodation and the cost and everything involved, it is massive and it (Parents)
Social opportunity	Breathe Magic creates social interaction among parents of a child with USCP	Enabler	3	Parents (3)	Yes	meeting other people, I think, because I’d never met another parent of a child with USCP or ... So that has been really important for us as well. (Parent)
	Parents wish social interaction could be continued long-term	Barrier	2	Parents (2)	No	I do want to keep in contact with people and see how they’re getting on, and you mentioned about learning to drive and that’s something that bothers me with Jasmine because, obviously, adapted cars and things like that don’t come easy, do they? So I just ... And jobs and things like that. So keeping that connection, maybe, long term rather than just short term. (Parents)
Reflective motivation	Breathe Magic makes therapy sound positive to talk about	Enabler	2	Parents (2)	No	friends and family ask you about Amelie and how she’s getting on, a lot of it is, “Oh, we’ve got this hospital visit and we’re going to do physio and we’re going to do this” and a lot of it’s quite negative, so it’s nice to have something quite positive to talk about. (Parents)
	Breathe Magic provides confidence to parents that their children can do things on their own in day-to-day activities	Mechanism	2	Parents (2)	No	I am so confident now that he will be able to do things without asking for help (Parents)
	Breathe Magic still requires parents to keep reminding children to practice their hands	Barrier	1	Parents (1)	No	but, again, I’ve noticed if you don’t keep reminding him now, he’s not thinking to do it again and he will just not ... As you say, it’s easier not to, he won’t necessarily do it. (Parents)
	Breathe Magic allows parents to encourage less for children to practice their hands	Enabler	1	Parents (1)	No	I am not so heavy on “Come on, come on you need to do that” (Parents)
Automatic motivation	Breathe Magic gives parents the chance to see unexpected achievement from their children	Enabler	1	Parents (1)	No	I think for me, being surprised by the things she could do after hard work. (Parents)

The relative importance of the six domains of COM-B in terms of frequency and elaboration is depicted in Table 3. The results are presented below in order of the total number of themes identified in each domain.

**Table 3:** Rank Order of COM-B Component Importance

COM-B component	Frequency of occurrence (N = total number of times this theme was mentioned by participants)	Number of sub-themes identified (N = number of sub-themes)	Discord (i.e. themes that are mixed- both barrier/enabler)	Expressed importance
1.Reflective motivation	58	14	No	Yes
2.Social opportunity	44	11	Yes	No
3.Physical capability	39	3	Yes	Yes
4.Automatic motivation	34	9	No	Yes
5.Physchological capability	7	4	No	No
6.Physical opportunity	6	3	Yes	No

### Reflective motivation

There were 10 reflective motivation mechanisms of change cited. The most frequent, discussed by both children and parents, was improvement in self-confidence. This was accompanied by reported improvements in determination: “you know I can try and I can try. I might not get it, but I can still give it a go”. Parents also reported increased self-motivation amongst children, with children inspired to keep trying to do things without external prompting. Other reported improvements were noted in children’s self-esteem, improved child confidence and comfort in discussing USCP with others, increased willingness amongst children to try new things, and increased child agency. Some parents also reported that their child’s self-awareness to improve their hand function increased. Parents further highlighted improved optimism themselves about their child’s future, and increased confidence in their child’s ability (“I am so confident now that he will be able to do things without asking for help”).

A key enabler of these mechanisms was the fact that the programme was a positive form of therapy as enablers. One parent explained: “friends and family ask you about [child’s name] and how she’s getting on, a lot of it is, ‘Oh, we’ve got this hospital visit and we’re going to do physio and we’re going to do this’ and a lot of it’s quite negative, so it’s nice to have something quite positive to talk about.” Further, children reported that they would like to recommend to other children with USCP that they should participate (demonstrating a reflective belief in the benefits of the programme and enjoyment in participating). However, while some parents reported that they therefore needed to remind their child to practise their exercises less (which they cited as a personal enabler), other parents reported that they still had to remind their child to practise, which they found at times to be a barrier: “I’ve noticed if you don’t keep reminding him now, he’s not thinking to do it again and he will just not.”

### Social opportunity

Ten separate themes around social opportunity were identified. Seven of these were mechanisms of change, with the most frequently cited being the formation of new friendships. This reportedly helped with integration at school following the programme, with one child becoming “the cool kid” having participated in the programme. Other mechanisms included the fact that the environment was relaxed as this made it easy for children to interact with new people and develop friendships, the strength and diversity of the learning environment (in particular the skill of the team and magicians), the nurturing team spirit of the programme, the strength of the peer support, the social environment which reduced feelings of loneliness in children, and the a holistic environment that was promoted by the programme, all of which encouraged child engagement.

Two factors were identified as enablers of this change. For children, the follow-up sessions offered opportunities for maintaining and strengthening the friendships made. For parents, having new parent support networks through the programme supported their engagement. However, two parents spoke about the challenge in continuing friendships after the end of the programme, especially when there were geographical barriers to meeting up.

### Automatic motivation

Children reported eight mechanisms of change relating to automatic motivation, including increased feelings of pride, decreased frustration, increased independence, decreased self-punishing behaviours, and improved feelings of helpfulness. Children also discussed how the camp staff tried to reinforce positive self-image: “They [Magic Camp staff] won’t do stuff for you. You have to do it all by yourself. Because they push you to feel good about yourself.” Children described the fun of learning magic: “This might seem a bit obvious, but just learning the magic and just practise it, practising, and trying and just learning new tricks. I like it all really. It’s a nice feeling just doing magic”. Participation also led to feelings of pride in their achievements: “It was kind of surreal because I was just like slowly progressing and I was watching myself do it”. Parents described the benefit of the non-medical approach for children’s motivation as a further mechanism of change: “that’s the important thing, isn’t it, that it’s therapy without being therapy”. Other mechanisms included the fact that parents reported children becoming less frustrated (“the frustration was reduced”), children becoming more independent, children discontinuing self-punishing behaviours, and children feeling more helpful. Parents also described the enabler for them of seeing their children achieve unexpected things: “being surprised by the things she could do after hard work.”

### Physical capability

With regards to mechanisms of change by which improved bimanual ability was achieved, the most frequently-reported was improvements in the child’s ability to perform physical daily tasks. This included children getting dressed, taking a shower, packing their own lunch for school, and cutting their food independently. As one child explained “Six months ago, I couldn’t tie a shoelace, now I can. Six months ago, I couldn’t even flap my hand. Now I can.” Further, another frequently reported mechanism of change was improved hand function for the child in relation to their affected hand. For example, children were able to use their affected hand more often: “I can do pinch and I can move my hand more”. However, a barrier to engaging in the intervention, expressed by both children and parents, is the experienced fatigue from taking part in Breathe Magic. For example, one parent reported that their child was too tired to leave and they needed to provide a snack to continue their journey back home. As another parent explained: “We would get some challenges sometimes, and she would be fatigued and I was exhausted.”

### Psychological capability

Two psychological mechanisms of change were highlighted for children: one parent spoke about their child’s increased independence, while one of the children reported being able to regulate their emotions better as a result of taking part: “I just used to go up to my bedroom sometimes every once in a while and just cry. I sometimes do that now, but I know how to get it better and stuff”. Additionally an enabler for parents was that they were able to take a step back and allow their child to become more independent.

### Physical opportunity

There were no mechanisms of change relating to physical opportunities identified. However, two physical opportunity barriers and enablers were raised as relevant to children. One parent mentioned the benefits of the initial camp being an intensive two-week programme for the children (enabler). However, one child disliked the follow-up clubs being on the weekend as it conflicted with other activities: “The thing I don’t like, it’s on a Saturday, it’s when I do my football” (barrier). Parents also identified two barriers/enablers relevant to them. They spoke about the location of the camp as either an enabler or a barrier depending on how close it was to where they lived: “when you have little...kids, it’s just too much. The journey just tired them out as well. So, if it’s a simpler journey it would be much easier for the child.” Further, for some who had not received funding to attend, the cost was reported as a barrier: “finding accommodation and the cost and everything involved, it is massive”.

## Discussion

This study applied the COM-B model to investigate the mechanisms of change in the Breathe Magic HABIT intervention for children with USCP by which an improvement in bimanual ability is achieved, and barriers and enablers to engaging in the intervention. This was explored from the perspective of both children participating in the intervention and their parents and/or carers. Mechanisms of change themes were identified within all the domains of the model, indicating that participating in Breathe Magic brings about change and positive outcomes by increasing capability, opportunity, and motivation. Additionally, a number of enablers to engaging in the intervention were identified, particularly under psychological capabilities, social opportunities and both reflective and automatic motivation. Very few barriers were raised; those that were raised were of relatively low frequency of reporting.

### Mechanisms of change

Regarding the mechanisms of change, the findings in this study show that two of the mechanisms (improved independent task performance and improved hand function in the affected hand) specifically related to Breathe Magic’s target of improving bimanual ability. These findings are consistent with existing quantitative research on improvement of motor function in children with USCP from the programme [19–21, 37]. Nevertheless, the reporting of these mechanisms does not imply that they are only achieved via Breathe Magic’s programme; such effects are also reported commonly from non-magic programmes [38, 39]. The positive and frequent reporting of these benefits suggests they provide a virtuous cycle of improvements leading to more engagement and effort, leading to more improvements. However, it is notable that these mechanisms relating to physical capabilities were in fact only the third most frequently reported mechanism of change. Other psychological, social and motivational factors also appear key to how and why Breathe Magic achieves its clinical benefits.

The most frequently discussed mechanisms of change by both parents and children were changes in reflective motivation as a result of participating in Breathe Magic. Previously, Green et al. (2013) found that magic-themed interventions deliver significant improvements in children’s self-confidence. Further, a qualitative study of parents whose children were involved in a magic-themed upper limb intervention reported that children felt ‘okay’ to be themselves as well as experiencing new feelings of motivation relating to improved self-belief [24]. This study builds on the findings from these two previous studies by identifying specific psychological benefits such as improved determination, self-motivation, self-esteem, self-awareness, self-determination, and optimism for the future. The improvements in self-motivation are particularly pertinent given motivation through goal setting (as is achieved in a magic programme where children have to master specific tricks) has previously been found to lead to improved motor function more than activity engagement without specific goals [18]. The improvements in self-determination are also key as children with USCP have been reported to give up on struggles quickly since they usually have to make a lot of effort even when conducting simple physical activities [40]. Breathe Magic instead encourages them to take on challenges, which may be a key factor in supporting improvements in physical capability. Children’s self-esteem was also identified as an important mechanism of change, with improved self-esteem being an enabler to ongoing engagement with the intervention. Self-esteem has a positive influence on children’s personal development [41], with higher self-esteem lessening the risk of children developing mental illnesses, committing crime, as well as improving physical well-being and economic prospects [42], and happiness and quality of life [43]. Many of these factors could be argued to be increased psychological capabilities. However, they were classified here as reflective motivators given that they not only led to improve psychological state in children but in turn led to a greater willingness to engage with the programme and in activities following the programme. These findings are of importance considering that a high number of children with USCP have psychiatric comorbidities [44], and that many other HABIT programmes do not have a dual focus on physical and psychological outcomes. Vincent-Onabajo, Lawan, Oyeyemi, & Hamzat (2012) [45] found a reciprocal relationship between children’s motor function (physical capability) and self-efficacy (reflective motivation). Based on this, it is possible that these two behavioural influences within the Breathe Magic intervention are mutually enhancing mechanisms.

Increased social opportunities were also highlighted as key mechanisms of change to engagement. For example, it was found that Breathe Magic provides an appropriate social environment to foster children’s peer relationships. These findings echo those from other studies that have identified the importance of socialising with other children with USCP in providing a sense of belonging, friendship, solidarity and joy [46, 47]. This is important given children with USCP have been reported to have lower levels of social maturity due to the increased dependence on parents [48], and children with poor peer relationships can have increased risk of adult psychopathology [49]. However, positive peer relationships and friendships can prevent children from developing behavioural and emotional difficulties [44, 50], provide a wide range of skills, attitudes and behaviour including social, listening, and emotional growth [51], and increase children’s motivation; an important factor in rehabilitation for people with USCP [52]. Breathe Magic also resulted in a reported improved integration of the participating children in mainstream schools. This is a notable finding considering that many children with USCP struggle with school life and commonly experience peer problems [51], and are more likely to get bullied or teased than children without USCP [53], which can lead to emotional stress or behavioural disturbances. The results presented here suggest that the Breathe Magic team, magicians, and mentors play an important role in nurturing these positive outcomes for children with USCP. Indeed, a systematic review of elements contributing to meaningful participation for children and youth with disabilities highlighted role models as a key factor [47].

### Does the ‘magic’ matter?

However, all of the mechanisms already reported are arguably common to multiple different programmes. Improvements in physical and psychological capabilities are widely reported across HABIT interventions, and similar physical opportunities are provided by other intensive therapy programmes [38, 39]. The social opportunities offered by group-based therapies for children with UCSP are also acknowledged by children and parents as key mechanisms [39, 54, 55]. Further, a number of CIMT studies have suggested that children can experience broader reflective motivational improvements as a result, such as improvements in self-confidence and determination [38, 39]. This all builds on broader literature on the key components for any interventions involving children with disabilities, which include developing friendships, having role models, learning, and developing one’s identity [47]. Nevertheless, there were several mechanisms that appear specific to the magic component of the Breathe Magic intervention.

First, in relation to automatic motivation, it was particularly highlighted that Breathe Magic focused away from medical interventions to fun, which appeared to motivate children to engage. This is important given that more than half of children with USCP have emotional difficulties such as anxieties and fears [44], suggesting the importance of interventions that help to reduce such negative psychological responses. Second, children reported that the magic itself was a fun skill. This echoes qualitative findings from interviews with parents from another magic-themed upper limb intervention that found that novelty and specialness of the magic approach was particularly enjoyable for children and their families and helped to increase self-belief and overall motivation [24]. Learning and having fun are key elements identified by the review on participation for children and youth with disabilities referenced above that are thought to contribute to meaningful participation [47]. Moreover, a study specifically focusing on children with USCP found that exploratory play was the activity of most interest to children [56], suggesting that Breathe Magic may target a particularly popular type of activity.

The enjoyability of activities has in fact been highlighted as critical for engaging children with USCP, with research showing that these children spend significantly more time engaging in activities of interest compared to those not of interest, and require significantly less physical assistance with such activities [56]. Automatic motivation in general is important to interventions for children with USCP as it associated with increased occupational performance [57, 58]. There are further mechanisms that have not previously been discussed in relation to other interventions for children with USCP and may be unique to magic-themed interventions (or potentially even to Breathe Magic). These include the holistic environment and good learning environment created by the magicians who lead the programme, the team spirit, and the reinforcement of positive self-image. However, these remain to be tested further to ascertain to what extent they really are unique. Therefore, although we lack a comparable study that has mapped all of the mechanisms activated by other interventions for children with USCP, it is clear that many of the mechanisms reported here from Breathe Magic are common to other programmes and are reported across studies of such programmes. However, the ‘fun’ focus of Breathe Magic and the magic element in particular appear well targeted to the demographic. Further studies exploring whether these elements may help to reinforce the other mechanisms are encouraged.

### Barriers and enablers

This study also had the secondary aim of identifying factors that could act as barriers or motivators to the mechanisms of change outlined above. With regards to motivators, many of these were factors that motivated parents to support their child’s engagement. Parents spoke about their children becoming less dependent on them and not needing to be reminded to practise using both hands before, which encouraged them to continue with the programme. Parents also discussed how the structure of the programme acted as an enabler, with the combination of two weeks of intensive camp followed by follow-up camps providing positive structure and supporting friendships both amongst the children and the parents. The fact that the programme was a positive version of therapy also acted as an enabler. However, this study also identified some barriers that could hinder the impact of, and engagement with, Breathe Magic. Notable barriers clustered around two of the COM-B domains: physical capability and physical opportunities. First, difficulties in accessing the Breathe Magic venue and cost of attending were highlighted. However, these only applied to some parents who lived further away or who were self-funding their places and are commonly reported across camp-based interventions for children with USCP [39]. Nevertheless, it is therefore recommended that the programme is run in different geographical locations to support ease of access for families, and that more funded places are made available for children to attend, including associated costs such as accommodation if required. Secondly, children and parents reported fatigue as a result of the intensity of the programme. While the programme follows a clinical model that precludes shortening the intervention, it is possible that addressing the issues around access and transport could reduce the reported fatigue.

### Strengths and limitations

Despite recommendations for the use of the theory in process evaluations [25], a recent systematic review identified infrequent and limited use of theory in process evaluations of healthcare interventions [28]. Therefore a strength of this process evaluation study is the use of a theoretical model of behaviour change as a framework to help structure analysis and interpretation of the data. Theory provides a generalizable framework through which to study mechanisms of change and factors influencing behaviours. However, a corresponding limitation of this study is that the interview topic guide was structured broadly around physical, psychological and social changes rather than around the domains of the COM-B model (although the use of COM-B as the framework for analysis was chosen prior to any analysis taking place). The interview questions were therefore not specifically geared towards eliciting themes related to the influence of capability, opportunity and motivation. Responses were thus spontaneously generated, and it may be that more themes within capability, opportunity and motivation could have been elicited if directly questioned about the potential role of each domain. Nevertheless, the interview prompts used were broad, and rich data and themes were generated from the focus groups and interviews. Relatedly, in this study we used qualitative interview and focus groups to identify mechanisms. However, methods require reflection (i.e. that individuals reflect on what is influencing their behaviour), and this might not always be a conscious process. Therefore, future studies that use alternative approaches such as ethnographies, other observational methods and quantitative assessments that prompt individuals to consider specific mechanisms are also welcomed.

In addition, although we follow guidelines for behavioural research in quoting the frequency of themes [33, 34], the elaboration of themes and the expressed importance, it is recognised that such metrics are not an objective marker of the importance of specific mechanisms and should be interpreted instead as giving indications as to the importance of identified mechanisms. In terms of potential source of bias, the high levels of inter-rater reliability indicate that there was a high level of agreement between two coders and reduced risk of subjective bias during analysis. However, the children and parents self-selected to participate in the interviews and focus groups. Although we had very high uptake, we approached potential participants in the final six month follow-up session, so whilst this has very high attendance from the original participants, it is possible that our sample was biased towards children and families who had experienced benefits from the intervention. Similarly, the nature of how the self-reported data was collected from the children and their parents might have caused social desirability bias and led to inflated participants’ ratings of the effectiveness of Breathe Magic. Future research could explore the barriers not just amongst those families who choose to participate, but also a purposive sample of families who declined to participate to identify why only some families engage.

## Conclusions

In conclusion, by conducting a theory-based qualitative process evaluation, this study demonstrated the mechanisms of change behind the Breathe Magic HABIT intervention for children with USCP by which it achieves improvements in bimanual ability. Breathe Magic was found to be a well-structured combination of multiple different mechanisms of change. Overall, the success of Breathe Magic was observed through not only its intended mechanisms to enhance hand skills, but also through unintended psychological improvements in children’s hand function, as well as social and motivational benefits resulting from interaction between children and parents. Many of these mechanisms, such as those relating to bimanual engagement, social interaction, and improved reflective motivation, have been identified as important for any programme aiming to engage children with USCP. But this study also identified further mechanisms that appear unique to the success of the Breathe Magic intervention; in particular the non-medical focus and the use of magic itself. Further, this study identified a number of enablers to engagement with the intervention that may inform future or similar interventions with this population group. However, barriers to engagement were also identified, which may inform refinements to the content and/or mode of delivery of Breathe Magic, to improve future engagement. This study also illustrates a replicable and systematic method for exploring how and why interventions work that may be applied to other interventions in treating child USCP.

## Data Availability

The datasets analysed during the current study are available from the corresponding author on reasonable request.

## Appendix 1: Topic Guide

**Topic guide for children**

**Table:**

Domain	Sample question
Response to the Magic camps	Had any of you done any magic before coming on this camp? Had you watched any magic tricks? What has been your favourite thing about the Magic camps so far? How does it make you feel to learn a whole trick and perform it? What is the achievement you are most proud about since taking part in the Magic camp?
Perceived impact	Have you noticed any differences in what you can do each day with your helper hand? What are the things you normally find most difficult? Have you found them easier since doing Magic? Have you felt more confident since doing Magic?
Future	Are you going to carry on doing magic after the end of the camps? Would you recommend the camps to other children? What advice would you give another child with hemiplegia?

**Topic guide for parents**

**Table:**

Domain	Sample question
Opening Questions	How do you feel your child has developed since attending Magic? [a nice opening question to warm people up, followed by some more specific things below]
Functional Questions	Functionally what improvements have you seen? Has the way your child faces challenges changed since the camp? How much functional support do you have to give your child now? Has this reduced/changed? How do you feel the rate of progress compares to before Magic?
Psychological	How does your child react to not being able to do something? Can you describe your child’s general wellbeing since starting the camp? Have you noticed changes in previous behaviours such as anxiety/phobias/mood changes in your child? Do you feel your child is more/less vulnerable/strong in the face of challenges? Have you seen any improvements in your child’s attention span? Does he/she become frustrated as much?
Social	How independent do you feel your child is? Or how much do they require support? Have you seen any impact on your child’s communication? Have you already seen/do you think there will be any impact on your children’s behaviour either at school or home? Have you already seen/do you think there will be any impact on your children’s social interactions such as with siblings or making friends at school? Do you think Magic will lead to your child being treated better by other children at school?
Parental	Do you feel the benefits of Magic have extended to you as parents? Has this had an effect on your own wellbeing?
General	a. What has been the best thing to come out of your child attending the camp? b. How has Magic changed the way you think about your child’s future? c. What would you say to another parent of a child with hemiplegia who was considering attending the camp? d. Is there anything else you’d like to say?

## Abbreviation

HABIT: Hand-Arm Bimanual Intensive Therapy
